# Kinetics and Mechanism of Camptothecin Release from Transferrin-Gated Mesoporous Silica Nanoparticles through a pH-Responsive Surface Linker

**DOI:** 10.3390/pharmaceutics15061590

**Published:** 2023-05-25

**Authors:** Nicolás Jackson, Andrea C. Ortiz, Alejandro Jerez, Javier Morales, Francisco Arriagada

**Affiliations:** 1 Institute of Pharmacy, Faculty of Sciences, Universidad Austral de Chile, Valdivia 5090000, Chile; njacksonbaez@gmail.com (N.J.); alejandrojerez@uach.cl (A.J.); 2 Facultad de Medicina y Ciencia, Universidad San Sebastián, Lago Panguipulli 1390, Puerto Montt 5501842, Chile; andrea.ortizo@uss.cl; 3 Department of Pharmaceutical Science and Technology, Faculty of Chemical and Pharmaceutical Sciences, Universidad de Chile, Santiago 8380494, Chile

**Keywords:** pH-responsive, mesoporous silica nanoparticles, camptothecin, kinetic, drug release

## Abstract

Stimuli-responsive nanomaterials have emerged as a promising strategy for inclusion in anticancer therapy. In particular, pH-responsive silica nanocarriers have been studied to provide controlled drug delivery in acidic tumor microenvironments. However, the intracellular microenvironment that the nanosystem must face has an impact on the anticancer effect; therefore, the design of the nanocarrier and the mechanisms that govern drug release play a crucial role in optimizing efficacy. Here, we synthesized and characterized mesoporous silica nanoparticles with transferrin conjugated on their surface via a pH-sensitive imine bond (MSN-Tf) to assess camptothecin (CPT) loading and release. The results showed that CPT-loaded MSN-Tf (MSN-Tf@CPT) had a size of ca. 90 nm, a zeta potential of −18.9 mV, and a loaded content of 13.4%. The release kinetic data best fit a first-order model, and the predominant mechanism was Fickian diffusion. Additionally, a three-parameter model demonstrated the drug-matrix interaction and impact of transferrin in controlling the release of CPT from the nanocarrier. Taken together, these results provide new insights into the behavior of a hydrophobic drug released from a pH-sensitive nanosystem.

## 1. Introduction

Cancer remains one of the major public health problems worldwide, with a high mortality rate, among various pathologies affecting the population [1]. Treating solid tumors with chemotherapeutics presents a challenging task, as it often leads to serious adverse effects due to the high doses administered, multidrug resistance, and low specificity [2,3]. In the last decades, significant advances have been made in the application of nanotechnology in biomedical and pharmaceutical sciences, primarily through the use of nanoparticles as drug delivery systems (DDS). This approach has shown promise in enhancing the pharmacokinetic profile of anticancer drugs [4,5,6,7]. Mesoporous silica nanoparticles (MSN) have received significant attention due to their porous structure and large surface area, which enables them to protect, transport, and release drugs [8,9]. In addition, their surface can also be chemically modified, making them versatile in their applications [10]. Moreover, stimuli-responsive systems can be obtained through structural modifications, which aid in spatiotemporal release control [3,11,12]. An interesting approach is to utilize pH as a stimulus to trigger the release of anticancer drugs due to the differences in pH between the physiological medium (pH ~ 7.4) and the microenvironment of the tumor cell (pH ~ 4.5–7.0) [13,14,15]. Theoretically, a pH-responsive nanomaterial designed for chemotherapy applications can hold a drug inside it without releasing it at pH 7.4, and only when it is in a more acidic environment, such as a tumor, can it release its cargo. Typically, the above is achieved by using MSN with a ligand on its surface that binds to a gatekeeper, which can be organic or inorganic in nature, to prevent the drug from escaping. Some biomolecules, such as proteins like transferrin (Tf), can act as both gatekeepers and directing agents, targeting the system towards tumor cells. This last feature is mainly due to the transferrin receptor being upregulated on the surface of cancer cells compared to healthy cells. Several studies have shown that Tf is a promising candidate for this role [16,17,18]. Furthermore, to immobilize the protein on the surface, various pH-sensitive linkers based on bonds of different natures have been reported, which have a faster hydrolysis rate at acidic pH than at alkaline pH [19,20,21,22,23]. Among them, the imine bond has been used to incorporate it in the synthesis of ligands and structures sensitive to pH, mainly because of its fast and relatively simple formation under certain conditions, which involves an amino group and an aldehyde (or carbonyl) moiety [24,25,26,27,28]. Therefore, the above combination generates a gatekeeper attached to the surface of the MSN through a pH-sensitive bond. This will enable the cleavage of the ligand between the nanoparticle and the gatekeeper only when the nanocarrier is exposed to an acidic microenvironment, leaving the surface free for selective drug release. For instance, Saini and Bandyopadhyaya have developed a pH-responsive system that delivers gemcitabine to pancreatic cancer cells. To achieve this, they conjugated Tf onto the surface of mesoporous silica nanoparticles, which were previously coated with chitosan via an imine bond [29]. On the other hand, Llinàs M. C. et al. used a hydrazone group as a pH-sensitive linker and a polyethylene glycol chain to produce a dual doxorubicin/camptothecin release triggered by acidic pH. They attached doxorubicin to the outermost end of the chain, acting as a gatekeeper for the camptothecin molecules [30]. Based on the imine bond formation strategy, Tian Z. et al. synthesized aldehyde-functionalized mesoporous silica nanoparticles using a silane. Then, they demonstrated the formation of an imine bond between the aldehyde moiety on the surface of nanoparticles and the amino groups on the BSA protein [21]. In a similar vein, recently Zhang Y. et al. functionalized hollow mesoporous silica nanoparticles with albumin-conjugated folic acid as a targeting agent for cancer cells. They showed that the formation of an imine bond on the surface of the nanoparticles facilitated the attachment of albumin, which serves as a gatekeeper. This led to a dual-responsive release of methylene blue, and doxorubicin triggered by an acidic environment [31].

Despite the various advances in the drug delivery field and the use of nanosystems as adjuvants in cancer treatment, improving anticancer efficacy remains a challenge. The literature reports that even after reaching the cell, the nanomaterial must face intracellular barriers that affect its anticancer performance [32,33,34,35]. Some of these barriers include interactions with organelles, endosomal escape, and the variable times and mechanisms still unknown in the removal of nanoparticles from inside the cell [36,37]. This leads to considering a specific period of time in which the drug must be released from the nanosystem; in this way, understanding the factors that affect the release from these advanced pharmaceutical forms is crucial to improve its efficacy. In this sense, studying the release kinetics allows the optimization of the design and manufacture of nanomaterials intended for biomedical applications to modify their release profile according to the specific requirements and thus anticipate their potential biological behavior.

Based on the above, in this work, we synthesized MSN, which were amino-functionalized and subsequently modified with glutaraldehyde, to form an imine bond closest to the surface of the nanoparticle, while free aldehyde groups were present at the outermost end. Afterward, these free aldehyde moieties reacted with the amino moieties in the transferrin, allowing the conjugation of the protein through the formation of an imine bond. As a result, a nanosystem with a gatekeeper linked by a cleavable ligand at acidic pH was generated. Additionally, the nanosystem was loaded with camptothecin as a model anticancer drug. Camptothecin (CPT) is an alkaloid isolated from the Chinese native tree *Camptotheca acuminata* [38]. Although CPT has been demonstrated to have an interesting anticancer effect due to its interaction with the nuclear enzyme topoisomerase I [39], its clinical use is limited by its insolubility in water, low specificity, high toxicity, and instability at physiological pH [40,41]. Therefore, the use of CPT as a model hydrophobic drug to transport it and subsequently release it is of great interest in pharmaceutical and biomedical research.

Finally, the nanocarrier was characterized, and the CPT release was studied under two pH conditions: pH 7.4, simulating the known physiological environment, and pH 5.0, simulating the tumor microenvironment. To better understand the release mechanism, the data were analyzed using five mathematical models and a three-parameter model that takes into account the drug interaction with the nanomaterial matrix.

## 2. Materials and Methods

### 2.1. Materials

(S)-(+)-Camptothecin (CPT ≥ 90%), human holo-Transferrin (Tf, ≥98%), tetraethyl orthosilicate (TEOS, 98%), (3-aminopropyl)triethoxysilane (APTES, ≥98%), cetyltrimethylammonium chloride solution (CTAC, 25 wt.%), ammonium hydroxide solution (acs reagent, 28–30%), and glutaraldehyde solution (GLUT, 25 wt.%), were purchased from Sigma-Aldrich. Ethanol (EtOH, HPLC grade), dimethyl sulfoxide (DMSO, for analysis), and hydrochloric acid (HCl, ACS reagent, fuming, 37%) were obtained from Merck. All other reagents and solvents were of analytical grade. Deionized water (Milli-Q, 18.2 MΩ·cm) was used in all experiments. All materials were used without any further purification.

### 2.2. Characterization

The hydrodynamic diameter was measured by dynamic light scattering (DLS) at 25 °C, using a Malvern Zetasizer Nano ZS90 (Malvern, UK) with a detection angle of 173° and an equilibration time of 120 s. The zeta potential was determined using the same equipment. Each sample was diluted in water at 25 °C, and the measurement was performed three times. The morphology and porous structure were verified using transmission electron microscopy (TEM) on a LIBRA 120 (Carl Zeiss, Oberkochen, Germany) model microscope with an accelerating voltage of 120.00 kV. Samples were prepared by dripping the suspension of nanoparticles in dry ethanol onto Formvar/carbon-supported copper grid (300 mesh) and allowing the sample to dry before measurement. N_2_ adsorption-desorption isotherms were measured at −196 °C using Micromeritics 3-Flex instrument (Micromeritics Instruments, Norcross, GA, USA). The specific surface areas and the pore volume were determined by Brunauer-Emmett-Teller (BET) and the Barret-Joyner-Halenda (BJH) methods, respectively. Thermogravimetric analysis (TGA) was performed on a Netzsch TG 209 F1 (Netzsch-Gerätebau GmbH, Selb, Germany) under an airflow of 20 mL min^−1^ with a heating rate of 10 °C min^−1^ in a temperature range of 30–800 °C. The physical state of the samples was studied by differential scanning calorimetry (DSC) on a SETARAM 131 Evo apparatus (SETARAM Instruments, Caluire, France). The samples were accurately weighted and sealed in aluminum pans. The pan was heated from 30 °C to 300 °C at a heating rate of 10 °C·min^−1^. Fourier-transform infrared (FT-IR) spectra were recorded using a Nicolet iS5 instrument (Thermo Scientific, Waltham, MA, USA) with 4 cm^−1^ resolution in the wavelength range of 4000–650 cm^−1^ averaging 16 scans. UV spectra were recorded on a Genesys 180 UV-vis spectrophotometer (Thermo Scientific, Waltham, MA, USA).

### 2.3. Mesoporous Silica Nanoparticles (MSN) Preparation

MSN were prepared by a modified Stöber method [42,43] in a hydroalcoholic medium. Briefly, 200 µL of NH_4_OH were mixed with a solution containing 65 mL of water, 12 mL of ethanol, and 7.94 mmol of CTAC at room temperature. The temperature of the mixture was raised to 80 °C under constant stirring, and after 45 min, 32.9 mmol of TEOS were added dropwise, allowing the reaction to proceed for 2 h at 80 °C. The suspension was centrifuged (13,000 rpm for 20 min) and washed with ethanol and water three times. The surfactant was removed by the solvent extraction method using an ethanol/HCl mixture for 12 h at 80 °C; the process was repeated twice. The MSN were obtained by centrifugation, washed thoroughly (ethanol and water), and dried under vacuum.

### 2.4. Amino-Functionalization and Aldehyde-Modified Surface

Amino-functionalized nanoparticles were obtained by modifying reported procedures [44]. Briefly, 340 mg of MSN were suspended in ethanol, and 4.1 mmol of APTES were added dropwise, allowing the reaction to proceed overnight at 40 °C. The nanoparticles were collected by centrifugation (13,000 rpm for 20 min), washed with ethanol, and denoted as MSN-NH_2_. Subsequently, the nanoparticle surface was aldehyde-modified using glutaraldehyde, adapting previous protocols [45,46,47,48]. To this, 100 mg of MSN-NH_2_ were suspended in 24 mL of PBS (0.01 M, pH 7.9), and 34 µL of glutaraldehyde were added. After stirring for 30 min at room temperature, the suspension was centrifuged (13,000 rpm for 20 min) and washed with PBS. The obtained product was denoted as MSN-CHO; thus, an imine bond (acid-labile) between the -NH_2_ group (nanoparticle) and the -C=O group (glutaraldehyde) was formed.

### 2.5. Transferrin Conjugation on the Surface of the Nanoparticles

Mesoporous silica nanoparticles were gated using Tf by the formation of an imine bond between the free-carbonyl moiety on the surface of nanoparticles and the amino groups in the Tf. To carry out the conjugation, a suspension of MSN-CHO was added dropwise to a solution of 2 mg/mL of Tf in PBS; afterward, the mixture was stirred for 24 h at room temperature. The final product was obtained by centrifugation, washed with PBS, and dried under a vacuum for further analysis. The conjugation efficiency (%CE) of Tf on the MSN was determined by TGA.

### 2.6. Drug Loading

The MSN-NH_2_ were suspended in a CPT solution (1 mg/mL) with a nanoparticle:drug mass ratio of 1:1 using DMSO as solvent. The mixture was stirred for 24 h at room temperature and then centrifuged. The supernatant was collected for quantification by UV-Vis at 366 nm. The drug loading content (%LC) and the drug loading efficiency (%LE) were calculated according to Equation (1) and Equation (2), respectively:

(1) $$\%LC=\frac{{CPT}_{total}-{CPT}_{free}}{Mass\;of\;final\;nanosystem}\times 100$$

(2) $$\%LE=\frac{{CPT}_{total}-{CPT}_{free}}{{CPT}_{total}}\times 100$$

where CPT_total_ and CPT_free_ are the total amount (mg) of CPT initially added to the mixture and the amount (mg) of free CPT in the supernatant, respectively.

### 2.7. In Vitro Release Profile

To assess the release profile of CPT, two types of media were used: PBS pH 7.4 (0.01 M) and acetate buffer pH 5.0 (0.01 M). As CPT has poor water solubility [49], DMSO was used as a co-solvent (5:28 *v*/*v*) following reported and validated protocols [30,50]. The experiments were carried out at 37 °C in a shaking incubator, following established procedures [51]. At each designated time point (1, 2, 3, 4, 5, 6, 12, and 24 h), the suspension was centrifuged, and the resulting supernatant was measured by UV-Vis at 366 nm. Fresh medium at 37 °C was used to replace the withdrawn volume, and sink conditions were maintained throughout the study.

### 2.8. Kinetics Analysis

Different mathematical models have been used to analyze or predict the release kinetics of a drug from different matrices or the dissolution of the drug from conventional pharmaceutical dosage forms [52,53]. Moreover, these models have proven to be effective in the analysis of drug release from nanomaterials [54,55,56]. The release data were analyzed according to the following kinetics models: zero-order (Equation (3)), first-order (Equation (4)), Higuchi (Equation (5)), the power law (Equation (6)), and the Peppas-Sahlin (Equation (7)):

(3) $$\frac{Q_{t}}{Q_{inf}}=k_{0}t$$

(4) $$\frac{Q_{t}}{Q_{inf}}=100(1-e^{-k_{1}t})$$

(5) $$\frac{Q_{t}}{Q_{inf}}=K_{H}t^{0.5}$$

(6) $$\frac{Q_{t}}{Q_{inf}}=K_{KP}t^{n}$$

(7) $$\frac{Q_{t}}{Q_{inf}}=K_{1}t^{m}+K_{2}t^{2m}$$

where Q_t_ and Q_inf_ are the amount of drug released at time = t and at time = infinite, respectively. k_0_, k_1_, K_H_, K_KP_, K_1_, and K_2_ are release apparent rate constants in each model. While n and m are the diffusional exponent and the purely Fickian diffusion exponent, respectively.

To demonstrate the impact of the gatekeeper on drug release, the release of CPT from Tf-free MSN-NH_2_ at pH 5.0 and pH 7.4 was also evaluated. The comparison of the release profiles was carried out by the similarity factor f_2_ according to Equation (8):

(8) $$f_{2}=50\log\left\{{\left[1+\frac{1}{n}\sum_{n=1}^{n}{\left({CPT}_{7.4}-{CPT}_{5.0}\right)}^{2}\right]}^{-0.5}\times 100\right\}$$

where CPT_7.4_ and CPT_5.0_ are the mean % of released CPT from the nanomaterial at pH 7.4 and pH 5.0, respectively, and n is the number of time points.

### 2.9. Statistical Analysis

The GraphPad Prism software version 8.0.1 (San Diego, CA, USA) and the DDSolver^®^ add-In (Microsoft Excel) program version 1.0 were used. To determine which model best fits the data, the coefficient of determination (R^2^) and Akaike Information Criterion (AIC) were used. The results are expressed as mean ± SD of three independent experiments.

## 3. Results and Discussion

### 3.1. Fabrication and Characterization of Tf Gated Mesoporous Silica Nanomaterials

**Figure 1:**
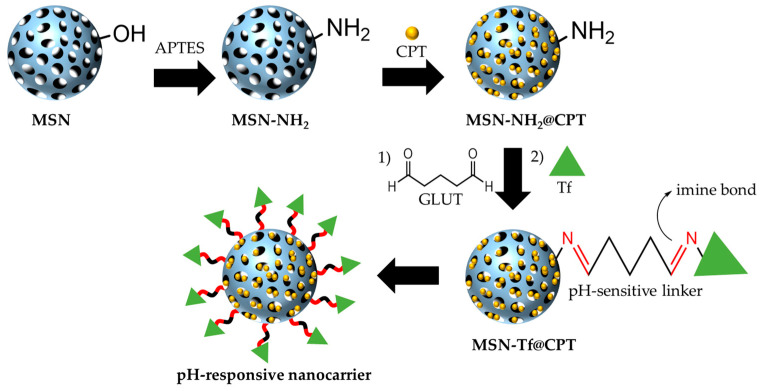
Schematic representation of the synthesis route of mesoporous silica nanomaterials. MSN: mesoporous silica nanoparticles; MSN-NH_2_: amino-functionalized mesoporous silica nanoparticles; MSN-NH_2_@CPT: camptothecin-loaded amino-functionalized mesoporous silica nanoparticles; MSN-Tf@CPT: camptothecin-loaded transferrin-gated mesoporous silica nanoparticles.

The MSN were obtained by a modified Stöber method, using CTAC as structure directing agent, and subsequently amino-functionalized onto the surface. Thus, -NH_2_ groups onto the surface can react with the carbonyl (C=O) group in the glutaraldehyde molecule to produce an imine bond (known as Schiff’s base). This bond has been widely studied as a linker in stimuli-responsive materials due to its higher rate of hydrolysis at acidic than alkaline pH [21,31,57]. After aldehyde modification onto the surface, the other carbonyl group at the end of the glutaraldehyde molecule is free to react with the amino groups of Tf, producing a new imine bond which finally attaches the protein to the nanoparticle surface (Figure 1). This produces a nanocarrier capable of transporting a drug (CPT), blocking its release at pH 7.4 (Tf as a gatekeeper) but releasing it in an acidic microenvironment (pH 5.0) due to the cleavage of the imine bond under such conditions.

**Figure 2:**
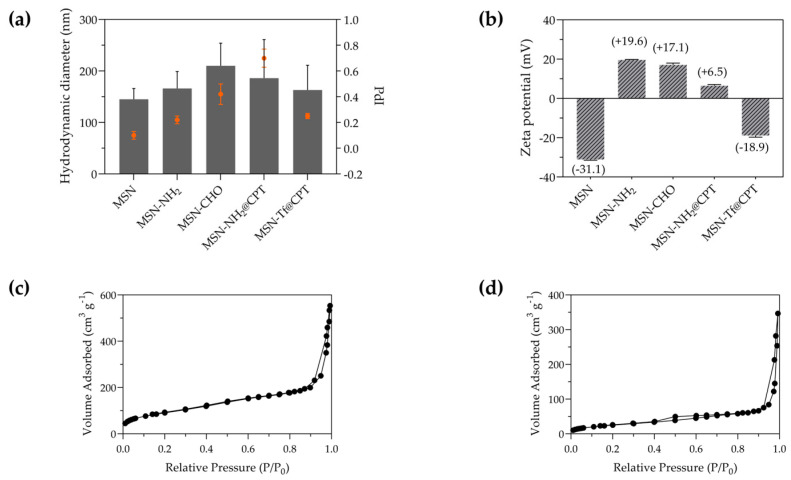
Physicochemical and physical characterization of the nanomaterials. (**a**) Hydrodynamic diameter (PdI on the right axis), (**b**) zeta potential, and N_2_ adsorption-desorption isotherms of (**c**) MSN-NH_2_, and (**d**) MSN-Tf.

The results obtained by DLS for the MSN show a hydrodynamic diameter of 145 nm (±21 nm) with a zeta potential of −31 mV (±0.3 mV) (Figure 2a,b). Upon modification of the surface, the MSN undergoes changes in its physicochemical properties. With amino-functionalization, the hydrodynamic diameter changes to 166 nm (±33 nm), and the zeta potential becomes +19 mV (±0.3 mV). Subsequently, with aldehyde functionalization, the size distribution increases, averaging 210 nm (±44 nm), while the zeta potential changes to +17 mV (±0.9 mV). The disturbance in the hydrated layers surrounding the nanoparticles explains the differences in the average size of the nanomaterials obtained by DLS. Furthermore, the change in zeta potential suggests successful amino-functionalization, while its subsequent decrease can be explained by the aldehyde modification due to the -NH_2_ and -C=O interaction.

The PdI values (Figure 2a) evidence the loss of colloidal stability and the aggregation of nanoparticles when the surface contains the carbonyl group from the aldehyde modification. After conjugation with Tf, the negative charge of the protein predominated, which gave suitable colloidal stability to the nanosystem.

**Figure 3:**
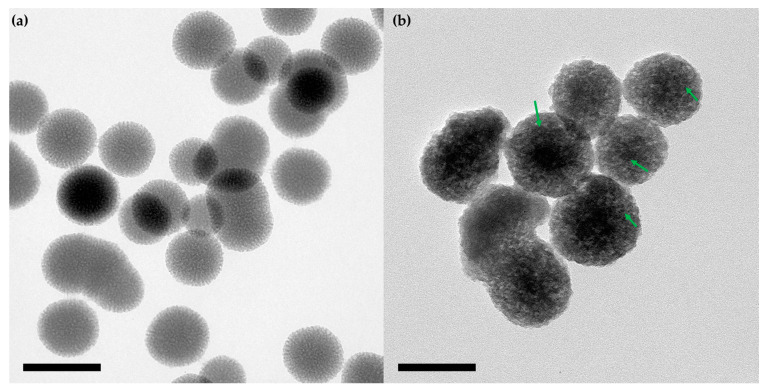
Morphological characterization of the nanomaterials. TEM images of the samples: (**a**) MSN and (**b**) MSN-Tf, scale bar: 100 nm. The green arrow highlights the dark spots as an example in each nanosphere.

Morphological analysis by TEM shows spherical nanoparticles with a size of ~90 nm (Figure 3a), which exhibited a porous structure with a well-defined regular edge [58,59].

The porous structure was confirmed by the nitrogen adsorption-desorption isotherm (Figure 2c,d), where the amino-functionalized mesoporous nanoparticle has a specific surface area of 333 m^2^·g^−1^ and a pore volume of 0.83 cm^3^·g^−1^ (Table 1). After the aldehyde functionalization and the Tf conjugation, the specific surface area and the pore volume decreased to 95 m^2^·g^−1^ and 0.51 cm^3^·g^−1^, respectively. This suggests that the grafting of the aldehyde groups and the protein conjugation occurred on the pore and surface of the nanoparticles. A TEM image of the Tf-gated mesoporous silica nanoparticles (Figure 3b) shows that the particles maintain their size, spherical shape, and porous structure. Moreover, dark spots were observed on the nanoparticles, which is attributed to the presence of transferrin [60].

**Figure 4:**
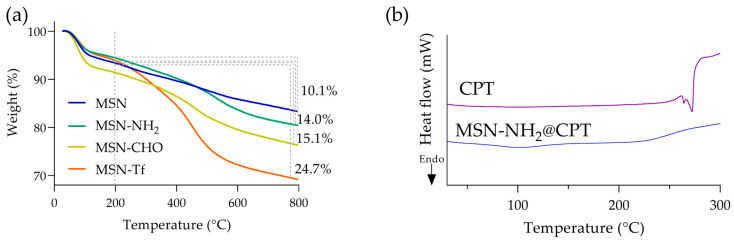
Thermal analysis of the samples. (**a**) Thermogravimetric analysis (TGA), and (**b**) differential scanning calorimetry (DSC).

The mass loss % of the samples was studied by thermogravimetric analysis. The mass loss of silica nanoparticles at temperatures above 200 °C is commonly attributed to organic matter and is correlated to the degree of surface functionalization [61]. The thermograms (Figure 4a) reveal the different extent of the surface functionalization, showing that the attached Tf content was 9.6%, which corresponds to 106 mg·g^−1^. Together the results indicate that protein has been effectively conjugated onto mesoporous silica nanoparticles. FTIR analysis was carried out to verify that the conjugation occurred through imine bond formation, which generates a linker that is pH-sensitive.

**Figure 5:**
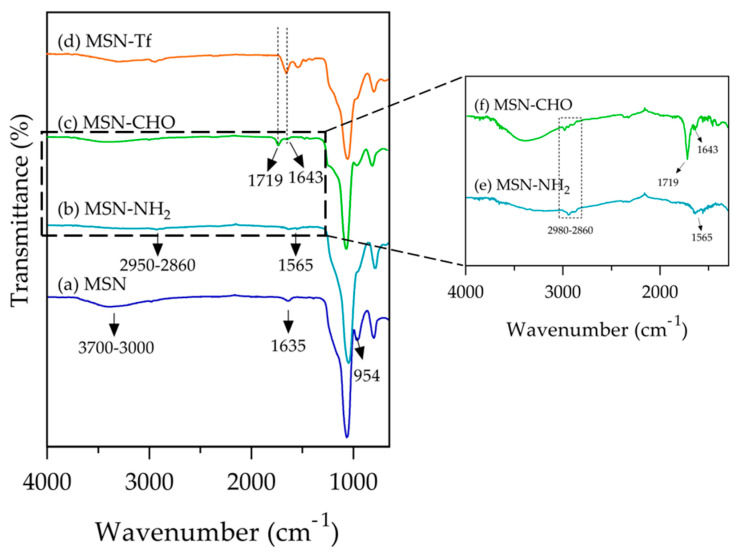
Fourier-transform infrared (FT-IR) spectra of the nanomaterials. (**a**) MSN, (**b**) MSN-NH_2_, (**c**) MSN-CHO, (**d**) MSN-Tf, (**e**) MSN-CHO zoom, and (**f**) MSN-NH_2_ zoom.

The MSN spectrum (Figure 5a) shows a typical band at 954 cm^−1^, 1067 cm^−1^, 1635 cm^−1^, and a wide band at 3000–3700 cm^−1^, attributed to asymmetric vibration of Si-OH, asymmetric vibration of Si-O, scissor bending vibration of molecular water, and O-H stretching (assigned to H-bonded water and silanol groups), respectively [62].

The amino-functionalization with APTES (Figure 5b,e) was verified with the presence of peaks at 2950 cm^−1^ and 2860 cm^−1^ corresponding to the stretching vibration of the C-H bond in the propyl chain of APTES. Additionally, the peak at 1565 cm^−1^ related to the N-H deformation modes was verified [63]. Figure 5c,f shows the spectrum of the MSN-CHO, where the peaks attributed to the -CH_2_ chain were observed in the region of 2860–2980 cm^−1^ due to the conjugation between GLUT and APTES. Moreover, the observation of a peak at 1719 cm^−1^, which is attributed to the free carbonyl group located at the end of the glutaraldehyde chain, provided evidence of the successful conjugation of the glutaraldehyde onto nanoparticles. The formation of the imine bond was evidenced by a distinct band at 1643 cm^−1^ [21,29]. In the FTIR spectrum of the MSN-Tf sample (Figure 5d), the peak assigned to the free carbonyl group in the aldehyde moiety disappeared, while the peak at 1643 cm^−1^ remained prominent, which is attributed to the C=N stretching vibration. Additionally, this peak overlaps with the characteristic bands of the Tf protein in the range of 1700–1300 cm^−1^, besides the peak at 3300 cm^−1^. The above confirmed that the protein attached to the nanoparticle surface through a linker containing imine bonds, which are pH-sensitive.

### 3.2. Drug Loading

The pores of MSN are capable of accommodating both hydrophobic and hydrophilic molecules, which is a key factor in their extensive application in the biomedical and pharmaceutical fields. Due to the hydrophobicity of the CPT, DMSO was selected as a suitable solvent to achieve the drug loading on MSN-NH_2_ [64] by an impregnation method. Following the incubation period, the supernatant was quantified, and the loaded CPT was calculated as 13.5% (LC) and 15.7% (LE) (Table 1). Consequently, the resulting CPT quantity in the nanomaterial was 0.45 mmol·g^−1^, which agrees with the range obtained in other reports under similar conditions [49,64,65,66]. After drug incorporation, the CPT interacts with the amino groups on the nanoparticles’ surface, which reduces the zeta potential and increases particle agglomeration. This is evidenced by the increase in the PdI beyond its ideal theoretical value (<0.3). However, after the intermediate synthesis steps, the protein was conjugated on the surface; at this point, the size measured by DLS decreases, the zeta potential becomes more negative (mainly because of the protein), and the PdI decreases dramatically. As a whole, colloidal stability is improved. After Tf conjugation, a small amount of CPT was lost during the washing procedures of the nanocarrier. Nevertheless, this quantity can be regarded as negligible because CPT is largely insoluble in water. Small fractions have been observed by other authors [67]. Additionally, the incorporation of CPT onto nanoparticles was evidenced by FTIR (Figure S1). The spectrum of Figure S1c shows four peaks that characterize CPT, that is, at 3429 cm^−1^, 1742 cm^−1^, 1654 cm^−1^, and 1600–1500 cm^−1^, related to the stretch of -OH, the ester group, the C=O, and the C=C vibrations in the rings, respectively [1,2,3]. The spectrum of Figure S1a (MSN-Tf) lacks these peaks, but they are present in the MSN-Tf@CPT sample (Figure S1b), evidencing the correct drug loading [64,68,69]. CPT loading onto MSN was also studied by DSC. The thermogram of pure CPT (Figure 4b) shows typical endothermic peaks at 264.16 °C and 272.34 °C, which corresponds to the melting temperature, evidencing the crystalline nature of CPT [70,71]. However, the curve corresponding to the nanoparticles loaded with CPT (MSN-NH_2_@CPT) did not present these peaks, evidencing the interaction between the CPT and nanoparticle, mainly within the pore, resulting in a noncrystalline state of CPT. The nanoparticle’s amino groups promote interaction within the pore, while the propyl chain provides a favorable hydrophobicity to CPT. It is expected that when the nanosystem is exposed to an aqueous medium, it will result in an improvement of the drug’s entry into the bulk solution.

### 3.3. In Vitro Release Profile

**Figure 6:**
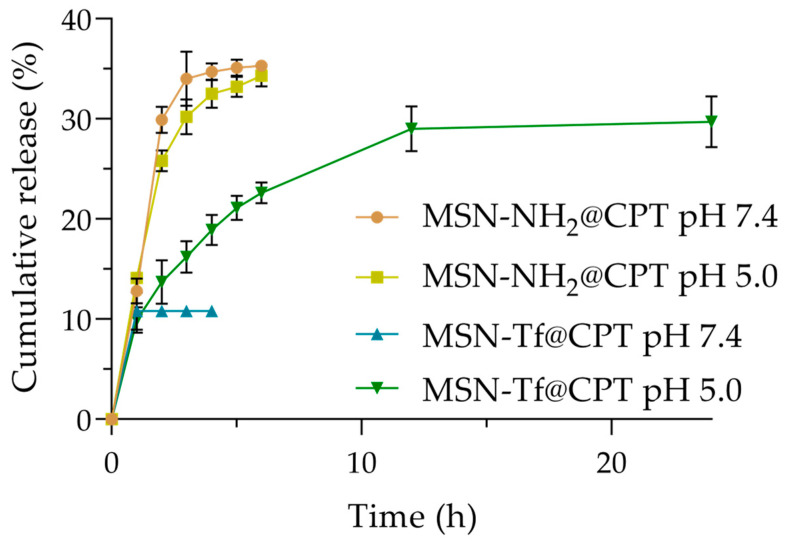
In vitro CPT release profile from different nanomaterials under pH 7.4 and 5.0 at 37 °C.

To evaluate the effect of the gatekeeper on the release of CPT, MSN-NH_2_ were used as a control nanosystem. The results show that the CPT release from the MSN-NH_2_ was pH-independent, releasing more than 30% of the loaded CPT in the initial 3 h (Figure 6).

After 6 h (~35% released) of the experiment, no CPT release was observed (Figure S2). This behavior can be mainly explained by two reasons. First, the absence of a key agent (such as a gatekeeper) that plugs the pore or coats the surface facilitates a rapid release. Hence, once the drug encounters the medium, both the dissolution process and the transport of the drug into the bulk solution begins. It can be observed that the release of CPT was slightly greater at pH 7.4 than at pH 5.0. This could be explained considering that at pH 7.4, the ionized form of CPT (carboxylate form) predominates; however, this form could interact with the positively charged amino groups under these conditions, thus affecting the release. Nonetheless, despite these factors, the differences are not significant, and both profiles are similar, as determined by the similarity factor (ƒ_2_ = 50.5). Secondly, the hydrophobicity of CPT and its null water solubility favor a high drug-matrix interaction, which limits the complete release from nanoparticles. This behavior has been described by other authors [49,51,72]. Conversely, the presence of Tf strongly impacts CPT release. Upon assessment of the MSN-Tf system at pH 7.4, a minor burst release of ca. 10% was observed (Figure 6), which is attributed to the drug adsorbed on the surface that is capable of diffusing. After this small amount was released, the nanosystem did not release any further CPT until the end of the experiment (due to the blocking effect of the gatekeeper), which would be a desirable characteristic in a potential application of a nanosystem targeting cancer cells. On the other hand, at pH 5.0, the rate of the hydrolysis of the imine bond is higher than at pH 7.4, producing the disassembly of the surface and leaving the pore free for the release of CPT. Contrary to the MSN-NH_2_ system, where the drug was released immediately, the release of CPT in the MSN-Tf system was prolonged, ultimately attaining a final value of 29.7%, which is comparable to the MSN-NH_2_ system. If we consider the amount of drug released by the control nanosystem (MSN-NH_2_), this is the maximum quantity that the nanoparticles could potentially release. In this way, it is shown that the release of CPT from the Tf-gated mesoporous silica nanoparticles is triggered by an acidic environment rather than an alkaline/neutral one due to the imine bond cleavage.

### 3.4. Kinetics Analysis

Five mathematical models were used to analyze the kinetic data to gain insight into the drug release mechanism. Although it is of interest to study the behavior of CPT release from the MSN-Tf system under various pH conditions, the release at pH 7.4 was excluded from the analysis because there was only one data point available. The reason for this is that the drug does not actually diffuse from the system, and the observed release is due to the small burst release of the weakly adsorbed CPT on the surface. The best-fitting model for the release data from the MSN-Tf at pH 5.0 was determined using both the coefficient of determination (R^2^) and the Akaike Information Criterion (AIC). To determine if the release follows a concentration-dependent or independent behavior, the zero-order and first-order models were compared. The data presented in Table 2 indicate that the first-order model provided a better fit than other models, indicating that the rate of release is concentration-dependent. This could be explained considering the nanosystem as a whole, where the surface does not remain constant due to the disassembly of the gatekeeper as a consequence of the fast hydrolysis of the imine bond at pH 5.0. In this process, a limited amount of drug slowly enters the solution, creating a gradient from which transport into bulk solution occurs. This behavior of CPT release from mesoporous silica nanoparticles has been observed by other authors [73]. It is expected that for a hydrophilic drug that has greater interaction with the surface of the nanoparticles under these pH conditions, the release profile will be affected, as well as the concentration-dependent behavior.

To investigate the mechanism by which the drug enters into bulk solution, three models were used. The data set fit the Higuchi model well (Table 3), suggesting that the CPT release from nanoparticles involves a diffusion process [74]. Moreover, the *n* diffusional exponent of the Korsmeyer-Peppas model was 0.46, suggesting anomalous (non-Fickian) transport (0.43 < n <1.0) [75]. It can be inferred from the results that there is more than one mechanism that controls the release, which makes sense considering that surface gatekeeper disassembly impacts drug-matrix interactions. Nevertheless, the value of the diffusional exponent is close to 0.43, which means that the release is likely to follow mainly Fickian diffusion. Moreover, the Peppas-Sahlin model has been useful in analyzing the contributions of coupled mechanisms through the constants K_1_ and K_2_. If K_1_ > K_2_, it is estimated that Fickian diffusion is the major mechanism in the release process [76]. The results show a purely Fickian diffusional exponent value of 0.45, and the kinetic constants of the model (K_1_ > K_2_) reveal that Fickian diffusion predominates in CPT release. It should be noted that the Korsmeyer-Peppas model provided the best fit for the data.

Furthermore, a three-parameter model was used to have a better understanding of the release mechanism considering the interaction between the drug and the matrix prior to the transport into the bulk solution. The model proposed by Zeng L. and Wu X. models the release considering that the reversible association and disassociation processes in the drug-matrix interaction (assuming that the drug molecules interact collectively with the system) follow first-order kinetics [77]. In this sense, the drug release is explained by the pore diffusion/convection of drug molecules from the porous matrix, as well as by drug-carrier interaction [78]. Equation (9) shows the three-parameter model:

(9) $$\frac{m_{t}}{m_{0}}=\frac{k_{off}}{\left(k_{on}+k_{off}\right)}\left(1-e^{-k_{s}t}\right)+\frac{k_{on}}{\left(k_{on}+k_{off}\right)}\left(1-e^{-k_{off}t}\right)$$

where k_off_ and k_on_ are the rate constant of the disassociation and association processes, respectively, while k_s_ is the diffusion/convection rate constant. m_t_ and m_0_ are the cumulative CPT release at time t and t = 0, respectively. In addition, the amounts of initially free and bound drug can be determined by the difference in free energy between the free and bound states expressed as $\Delta G=-k_{B}T\ln\left(\frac{k_{on}}{k_{off}}\right)$, where k_B_ is the Boltzmann constant (1.381 × 10^−23^ J), and T is the absolute temperature (310 K).

The results show that k_s_ >> k_off_ (Table 4), which indicates that the diffusion and the convection are not neglected during steady-state release [78]. Figure 6 shows that the CPT release from the Tf-gated nanoparticles does not achieve 100%, which indicates that the system tends to reach a partition equilibrium. This equilibrium is governed by factors such as the relative strengths of surface-drug and solvent-drug interactions, as well as the entropy of the system [79].

In this sense, the ΔG negative value suggests that the desorption is not favored, mainly due to the hydrophobicity of the CPT. Furthermore, the value of k_s_ provides quantitative information about the CPT diffusion rate during the burst release stage [80].

Here, the reported value (k_s_ = 0.34 h^−1^) shows a slow burst release. The impact of Tf on the drug release was observed through changes in both the burst release magnitude and the value of k_s_. All samples exhibited a ΔG with a negative value, but the system without Tf demonstrated a greater magnitude. While the disassociation process rate constant was similar among the samples (Table 4), the Tf-free nanosystem displayed a higher rate during the burst release stage (k_s_ = 0.67 h^−1^ and 0.65 h^−1^, at pH 7.4 and 5.0, respectively). This can be explained by the retentive effect of the gatekeeper on the surface and its ability to control the CPT release based on the hydrolysis rate of the imine bond triggered by an acidic environment.

## 4. Conclusions

We modified mesoporous silica nanoparticles by attaching transferrin on their surface via a linker that contains imine bonds. The main goal was to load CPT onto the nanoparticles and trigger their release in response to an acidic pH to then study the release kinetics. An evident enhancement in the apparent solubility of the CPT was observed upon loading it onto the amino-functionalized nanoparticles. Nanomaterial characterization confirmed that the imine bond was successfully formed on the surface of the nanoparticles, allowing for the conjugation of transferrin, which acted as a gatekeeper. This gatekeeper role ensured that only a minimal amount of CPT escaped from the nanosystem at pH 7.4, while at pH 5.0, a controlled release of CPT was achieved. From the kinetic analyses, we concluded that the CPT release data from the pH-responsive nanosystem best fit a first-order model, following an anomalous transport mechanism, but with Fickian diffusion as the dominant process. Furthermore, the utilization of the three-parameter model in the analysis validated the role of transferrin as a gatekeeper in controlling the release of CPT, as well as the influence of the drug-matrix interaction, which limited the release. Together, these results provide a deeper understanding of the release behavior of a hydrophobic drug from a pH-responsive nanosystem. This knowledge could guide the design of silica-based nanomaterials intended for biomedical applications to achieve a specific rate of drug release under acidic pH conditions.

## Figures and Tables

**Table 1:** Particle characterization and loaded parameters. Specific surface area (S_BET_), pore volume, loading content (%LC), loading efficiency (%LE), and conjugation efficiency (%CE). SD is the standard deviation (n = 3).

Nanoparticle	S_BET_ (m^2^·g^−1^)	Pore Volume (cm^3^·g^−1^)	%LC	%LE	%CE ^a^
MSN-NH_2_	333	0.83	-	-	-
MSN-NH_2_@CPT	-	-	13.5 ± 1.8	15.7 ± 0.9	-
MSN-Tf@CPT	95	0.51	13.4 ± 0.6	15.5 ± 0.2	9.6

^a^ Obtained by TGA.

**Table 2:** Kinetics parameters for the CPT release from MSN-Tf nanosystem.

Nanosystem	Medium	Kinetics Model					
		Zero-Order $\frac{Q_{t}}{Q_{inf}}=k_{0}t$			First-Order $\frac{Q_{t}}{Q_{inf}}=100(1-e^{-k_{1}t})$		
		k_0_	R^2^	AIC	k_1_	R^2^	AIC
MSN-Tf@CPT	pH 5.0	10.8	0.439	67.2	0.25	0.971	43.4

k_0_ (%·h^−1^); k_1_ (h^−1^).

**Table 3:** Kinetics parameters for the CPT release mechanism from MSN-Tf nanosystem.

Nanosystem	Medium	Kinetics Model									
		Higuchi $\frac{Q_{t}}{Q_{inf}}=K_{H}t^{0.5}$			Korsmeyer-Peppas $\frac{Q_{t}}{Q_{inf}}=K_{KP}t^{n}$			Peppas-Sahlin $\frac{Q_{t}}{Q_{inf}}=K_{1}t^{m}+K_{2}t^{2m}$			
		K_H_	R^2^	AIC	n	R^2^	AIC	K_1_	K_2_	R^2^	AIC
MSN-Tf@CPT	pH 5.0	30.4	0.983	38.9	0.46	0.999	3.4	32.5	0.73	0.999	5.0

K_H_ (%·t^−0.5^); K_1_ (h^−0.45^); K_2_ (h^−0.9^).

**Table 4:** Kinetics release parameters for the CPT-loaded nanomaterials, according to the three-parameters model.

Nanosystem	Medium	Kinetics Model			
		Three Parameters			
		k_s_	k_off_	ΔG (10^−21^)	R^2^
MSN-Tf@CPT	pH 5.0	0.34	0.0021	−5.93	0.984
MSN-NH_2_@CPT	pH 7.4	0.67	0.0022	−4.39	0.971
	pH 5.0	0.65	0.0017	−4.68	0.992

K_s_ (h^−1^); k_off_ (h^−1^); ΔG (J).

## Data Availability

The manuscript and supplementary material contain the reported data. Additional relevant data can be obtained upon request from the corresponding author.

## Supplementary Materials

The following supporting information can be downloaded at: https://www.mdpi.com/article/10.3390/pharmaceutics15061590/s1, Figure S1: FTIR spectra of nanomaterial samples and raw drug. (a) MSN-Tf, (b) MSN-Tf@CPT, and (c) CPT; Figure S2: Full release profile of camptothecin from nanomaterials.
