# Supplementary material for: Kinetics and Mechanism of Camptothecin Release from Transferrin-Gated Mesoporous Silica Nanoparticles through a pH-Responsive Surface Linker
Source: Pharmaceutics. 2023 May 25;15(6):1590. doi: 10.3390/pharmaceutics15061590 (PMC10303495; doi:10.3390/pharmaceutics15061590)
Supplement: Supplementary file 1 [file pharmaceutics-15-01590-s001.zip › pharmaceutics-2382542-supplementary.pdf]

Supplementary Materials: Kinetics and Mechanism of Camptothecin Release from Transferrin-Gated Mesoporous Silica nanoparticles through a pH-Responsive Surface Linker

Nicolás Jackson, Andrea C. Ortiz, Alejandro Jerez, Javier Morales and Francisco Arriagada

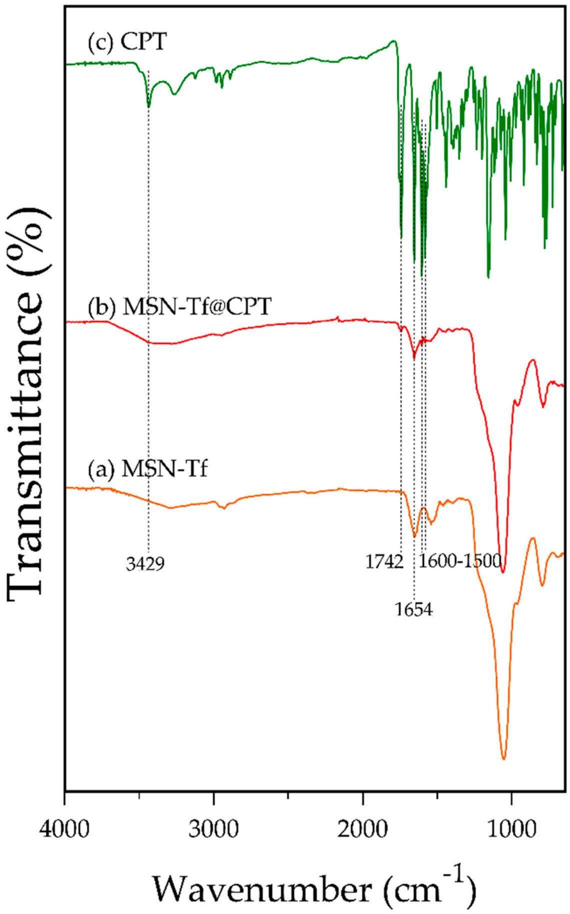

Figure S1. FTIR spectra of nanomaterial samples and raw drug. (a) MSN-Tf, (b) MSN-Tf@CPT, and (c) CPT.

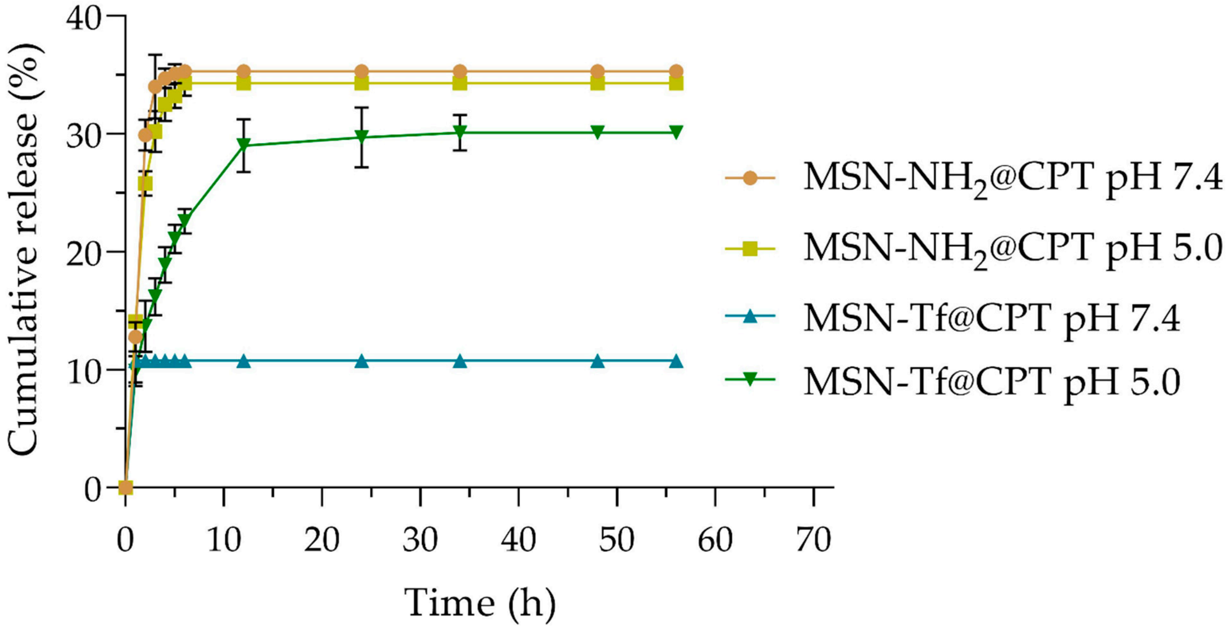

Figure S2. Full release profile of camptothecin from nanomaterials.
